# Deceased donor skin allograft banking: Response and utilization

**DOI:** 10.4103/0970-0358.70732

**Published:** 2010-09

**Authors:** Madhuri A. Gore, Anuradha S. De

**Affiliations:** Department of Surgery, Lokmanya Tilak Municipal Medical College and General Hospital, Sion, Mumbai – 400 022, India; 1 Department of Microbiology, Lokmanya Tilak Municipal Medical College and General Hospital, Sion, Mumbai – 400 022, India

**Keywords:** Benefits, deceased donor, response, skin allograft, utilization

## Abstract

**Background:**

In the absence of xenograft and biosynthetic skin substitutes, deceased donor skin allografts is a feasible option for saving life of patient with extensive burn injury in our country.

**Aims:**

The first deceased donor skin allograft bank in India became functional at Lokmanya Tilak Municipal (LTM) medical college and hospital on 24^th^ April 2000. The response of Indian society to this new concept of skin donation after death and the pattern of utilization of banked allografts from 2000 to 2010 has been presented in this study.

**Settings and Design:**

This allograft skin bank was established by the department of surgery. The departments of surgery and microbiology share the responsibility of smooth functioning of the bank. Materials and Methods: The response in terms of number of donations and the profile of donors was analyzed from records. Pattern and outcome of allograft utilization was studied from specially designed forms.

**Results:**

During these ten years, 262 deceased donor skin allograft donations were received. The response showed significant improvement after counselling was extended to the community. Majority of the donors were above 70 years of age and procurement was done at home for most. Skin allografts from 249 donors were used for 165 patients in ten years. The outcome was encouraging with seven deaths in 151 recipients with burn injuries.

**Conclusions:**

Our experience shows that the Indian society is ready to accept the concept of skin donation after death. Use of skin allografts is life saving for large burns. We need to prepare guidelines for the establishment of more skin banks in the country.

## INTRODUCTION

The tertiary referral burn centre established in 1983 at this medical college and hospital receives about 600 patients with burn injury annually. Protocol-based burn management with strict attention to resuscitation, infection control and nutrition was implemented since 1989 when the author was appointed as chief of burn centre. Development of banana leaf dressing in 1997[1] contributed to early healing of superficial partial thickness burn wounds. All these efforts resulted in the improvement of the survival probability from LA50 of 35% total body surface area (TBSA) burns in 1989 to 50% TBSA burns in 1997.

At this point, it was realized that for any further improvement in the outcome, availability of a substitute for autologous skin was essential. With non-availability of porcine xenografts and impossibility of obtaining biosynthetic skin substitutes, the need for a facility to bank cadaver skin allograft was acutely perceived. After the necessary permissions and preparations, the first deceased donor allograft skin bank in India became functional on 24^th^ April 2000. This article takes an overview of the efforts to introduce the concept of skin donation after death to Indian society, the response to this venture and the pattern of utilization of the cadaver skin allograft over the past ten years.

## MATERIALS AND METHODS

Permission for establishing a skin bank with procurement of deceased donor skin allograft was obtained from the State Appropriate Authority of the Government of Maharashtra. This was based on the Bombay Anatomy Act of 1949 and on the definition that though skin is an organ, split-thickness skin graft (STSG) is a tissue.

Protocol preparation: Initiation of skin banking involved preparation of several protocols including those for response to donation call and procurement of deceased donor skin allograft with consent of the next-of-kin. The protocols for quality control included microbiological studies and viability testing besides details of preservation methods.

Awareness campaign: Inauguration of this skin bank also marked the introduction of the concept of skin donation after death to the Indian society. The need for concentrated and continuous efforts to create awareness about this till then unheard of topic was recognized by the first author. Preparation of brochures, posters, flip charts and PowerPoint presentations augmented the articles in print media, television interviews and radio talks. This campaign is ongoing.

Microbiological data review: Analysis of the microbiology of deceased donor skin allografts was carried out on regular basis and appropriate modifications were made in the donor area cleaning protocol and graft procurement protocol.

Protocol modification and training: Till the end of year 2006, the donor areas were cleaned with combination of povidone iodine and quaternary ammonia compound. This practice was changed to use of 0.5% chlorhexidine solution followed by alcohol cleaning and application of 5% povidone iodine solution. The donor area cleaning was changed to sequential cleaning, and wiping of skin graft blade and handle with alcoholic solution after completing one area was introduced.

Training of all the general surgery residents about the method of deceased donor skin allograft procurement was introduced as a regular feature.

Cryopreservation at −70°C with 15% glycerol as cryoprotectant was used for the preservation of allografts from the year 2000 to 2006. Since 2007, high-concentration glycerol preservation method developed by Euro Skin Bank[2] is being utilized for majority of the allografts.

Review of response and strategy modification: At the end of the year 2006, the response to this newly introduced concept of skin donation after death was reviewed. With 56 donations in a six-year period, a modification in the counselling strategy was considered essential. Till then the counselling responsibility rested mainly with the consultants and residents of department of surgery. The counselling of the relatives was expected to be carried out after death of a hospitalized patient. This was obviously ineffective and the reasons were many. It was decided to extend the counselling outside the hospital in the community as well. Members of Sunday Friends – a voluntary organization – were provided all the information about this subject along with relevant photographs. A group of general practitioners also received training for counselling. Analysis of the profile of donors was performed once every year and issues related to skin procurement were discussed and sorted out every three months in a meeting of Sunday Friends with surgical residents, members of burn team and the microbiologist of skin bank.

Analysis of utilization: With improved availability of skin allograft, the utilization was streamlined with strict record keeping of requisition and allocation. The age, sex and burn extent of the recipients, the reason for use of allograft and the outcome was analyzed for the 10-year period. The duration of graft survival and issues related to handling of skin allograft were also studied.

No test of statistical significance was applied to this data.

## RESULTS

### Awareness campaign

This is an ongoing activity. The details of different methods adopted and their frequency over the ten years of existence of skin bank have been provided in Table 1. At present, the skin bank holds pledge forms of 732 individuals wishing to donate skin after death.

**Table 1:** Different methods of awareness campaign

*Methods of awareness campaign*	*Number*
Awareness talks	73
Articles, interviews in print media	32
Television interviews	9
Dial-in radio programmes and talks	7
Educational film	1

### Microbiology of skin allograft and impact of protocol modification

A study of pre-processing microbiology of skin allograft from 33 donors (years 2002−2006) revealed positive pre-processing cultures in 70%[3] with fungal growth in 23.3%. The micro-organisms most commonly isolated were *Escherichia coli*, *Staphylococcus aureus* and *Candida* spp. This high incidence of contamination led to modification of cleaning and procurement protocol as specified earlier. The frequency of positive pre-processing cultures was 20.39% in 206 donations received during subsequent period from Jan 2007 to 31^st^ March 2010 [Table 2] and the common isolates were *E. coli*, *Enterobacter* and *Enterococci*, Methicillin-sensitive *S. aureus* and *Candida*. species.

**Table 2:** Frequency of positive pre-processing culture in skin allograft

*Culture status*	*2007*	*2008*	*2009*	*Upto 31.3.2010*	*Total*
Positive	26	13	3	nil	42 20.39%
No growth	27	57	64	16	164 79.61%
Total	53	70	67	16	206 100%

### The response

During the 10-year period between 24^th^ April 2000 and 31^st^ March 2010, deceased donor skin allograft donation was received from 262 donors. Out of these, allografts from seven donors (2.67%) had to be discarded due to positive serology. The positive serology included three that tested positive for treponemal antibody (VDRL), two for HBsAg and one each for HIV and HCV. During this period, nine donations were regretted due to unsuitability of the proposed donor (known HBV, HCV, presence of jaundice and systemic sepsis). From 24^th^ April 2000 to 31^st^ December 2006, a total of 56 donations were received. Till then, the counselling was entrusted to clinicians and was mainly restricted to the hospital premises. From the year 2007, the counselling is being done mainly by members of a voluntary organization and a group of family physicians in the hospitals as well as in the community after a death occurring at residence. From 1^st^ January 2007 to 31^st^ March 2010, 206 donations have been received. This difference in the number of donations in these two time periods was striking.

Out of 262 donors, 154 (58.78%) were males and 108 (41.22%) were females.

The youngest donor was 16-year-old and the eldest was 104 years of age. The age distribution of donors is provided in Table 3. The largest numbers of donors (62.21%) were more than 70 years of age.

**Table 3:** The age distribution of donors

*Age groups*	*Number of donors*	*Percentage*
<40 years	35	13.36
40–70 years	64	24.43
> 70 years	163	62.21
Total	262	100

The skin allograft procurement was performed within four hours of death in 149 (56.87%) donors and the time lapse between death and graft procurement was more than four hours for 113 (43.13%) donors.

The details of location of allograft procurement have been provided in Table 4. Out of 158 donations that were procured at home, only five were during the period from 24^th^ April 2000 to 31^st^ December 2006. From 2007 onwards, majority of the donations that is 154 out of 206 (74.76%) have been received at home.

**Table 4:** Locations of allograft procurement

*Locations*	*Number of donations*	*Percentage*
Home	158	60.31
LTMG hospital	45	17.17
Other hospitals	59	22.52
Total	262	100%

The average size of the grafts procured from each donor was 1500 sq.cms.

Skin donation after death was associated with eye donation or body or organ donation in 123 (46.95%) donors. The most frequent association was of skin and eye donation in 65 (24.81%) donors. This association of donations is depicted in Table 5.

**Table 5:** Association of donations

*Association*	*Number of donors*	*Percentage*
Skin + eyes	65	24.81
Skin + eyes + body	28	10.69
Skin + body	26	9.92
Skin + organs	4	1.53
Skin only	139	53.05
Total	262	100

### Analysis of utilization

Till 31^st^ March 2010, skin allografts from 249 donors were utilized for 165 patients. Of these, allografts from 9 donors were provided for 7 patients (6 with burn injury and 1 non-burn patient) in four hospitals in the city other than our institution. Thus 158 patients from our institution received allografts from 240 donors. Four recipients had soft tissue loss due to trauma and three had necrotizing fasciitis. Skin allografts from eight donors were utilized for these seven patients. Most of the recipients that is 151 (95.57%) had burn injury and received allografts from 232 donors. Different indications for which allografts were utilized in burnt patients have been detailed in Table 6.

**Table 6:** Indications for allograft utilization in burnt patients

*Indications*	*Number of patients*	*Percentage*
Primary excision and wound closure	48	31.79
Promotion of epithelization	37	24.50
Poor general condition of the patient	23	15.23
Infection control and wound bed preparation	43	28.48
Total	151	100

The distribution of recipients according to the burn extent and their outcome along with the number of allograft donors is provided in Table 7.

**Table 7:** Burn extent, allograft utilization and outcome

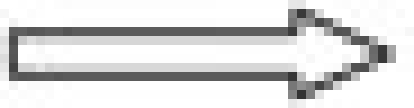	*< 30%*	*30 – 50%*	*> 50%*	*Total*
*Burn extent*	*TBSA*	*TBSA*	*TBSA*	**
No. of recipients	9	58	84	151
	5.96%	38.41%	55.63%	100%
No. of survivors	8	56	80	144
No. of deaths	1	2	4	7
No. of donors	8	76	148	232
	3.45%	32.76%	63.79%	100%

Nine patients in paediatric age group with full-thickness burn extent of 11−23% TBSA received skin allografts. Allografts were utilized, since procurement of autografts was considered inappropriate due to poor nutritional status, presence of invasive burn wound sepsis and poor general condition of the patients. The youngest patient was three years of age. All these patients were received from other healthcare facilities after variable treatment period. There was one death in this group [Table 7]. All the remaining patients (142) were young adults with age ranging from 19 to 37 years. Of these seven were males and 135 were females. Seven (4.64%) out of 151 recipients of allografts expired. Four succumbed to overwhelming sepsis, two due to poor general condition with severe anaemia and hypoproteinaemia and one due to multi-organ failure following primary excision. The survivor with largest burn extent had sustained 85% TBSA burns in a burn mass casualty incident.

The duration of allograft survival on the recipient ranged from 6 to 31 days with median of 16 days. No problems were encountered in utilization of either cryopreserved or glycerol-preserved allografts.

## DISCUSSION

Skin allografts were first used, at the end of the last century (1881), by Girdner. However, the first proper skin bank was the US Navy Skin Bank, set up in 1949.[4] Overcoming all the hurdles, discouragements and difficulties, the first deceased donor skin allograft bank in India became functional at LTM Medical College and General Hospital, Sion, Mumbai on 24^th^ April 2000.

In most countries in the world, skin donation is linked with organ donation programme and hence comprehensive counselling is undertaken. Unfortunately, such program does not exist in our country till today. Hence, creating awareness about this complete new concept of skin donation after death was a significant challenge. Several methods were adopted for this campaign [Table 1] and today the skin bank has more than 700 registered donors. This campaign has to be an ongoing process. Search has failed to reveal any published article related to the awareness campaign for the promotion of cadaver skin donation.

The importance of continuous quality control measures cannot be overstressed. An interim analysis of pre-processing microbiology of the skin allografts (years 2002−2006) revealed 70% incidence of positive cultures in 30 donations. With appropriate modifications in the allograft procurement protocol, the frequency of positive cultures decreased to 20.39% in 206 donations [Table 2] received during the subsequent period (years Jan 2007− March 2010). This compares well with the most recently reported frequency of 26.55% (192/723) from Siena Skin Bank, Italy.[5]

In the year 2007, the method for allograft preservation was changed to predominantly glycerol preservation from predominantly cryopreservation. Problems in maintenance and repairs of ultra-cool refrigerator, non-availability of uninterrupted power supply despite several efforts and lower cost of the method were the main reasons for this change in the protocol. Glycerol preservation has been shown to be effective[6] and significant data is available regarding satisfactory utilization of glycerol-preserved skin allografts.[7–9] Our experience of use of glycerol-preserved allografts has been extremely satisfactory too. Newer methods of preservation using high concentrations of propylene glycol[10] and disinfection with peracetic acid and preservation in glycerol[11] have also been described, but we have no personal experience to share.

The burn care professionals in India have generally been sceptical and cynical about the acceptance by and attitude of Indian society towards this recently introduced concept of skin donation after death. But our experience has been encouraging. We have received 262 donations in 10-year period. Siena Skin Bank in Italy[5] (723 donors in 7 years) and Euro Skin Bank have reported a better response. We have received more donations than Japan (214 in 11 years).[12] Detailed profile of the donors was not available in literature. Our analysis of 262 donors shows that we have more male donors (154 i.e. 58.78%). Allografts from 7 (2.67%) donors had to be discarded due to positive serology for transmissible diseases. This discard rate though low, underscores the importance of detailed medical history and screening of the proposed donor. Majority that is 56.87% of the procurements have been within four hours of death indicating the promptness of response. Procurements after four hours after death have been associated with body donation 50% of the times. As skin donation is a part of multi-organ donation programme, donors more than 70 years of age are not accepted by most of the skin banks in the world. We do not observe any upper age limit for donors and our eldest donor was 104 years of age. In fact, 62.21% of our donors were more than 70 years old [Table 3]. Procurement of skin grafts was technically challenging in these elderly donors. However, we have not observed any inferiority in the efficacy of these allografts. After extending the counselling to the community, allograft donation was procured at the residence of the deceased on 158 (60.31%) occasions [Table 4]. Non-availability of a dedicated vehicle for transport did cause difficulties, but the response team of general surgery residents managed to overcome the hurdles successfully with determination and dedication. Our data shows [Table 5] that 123 out of 262 (46.95%) skin donations were associated with donation of eyes, body or organ, the commonest being eye donation. It is necessary that all different organizations working to promote deceased donor organ and tissue donation must come together to implement a comprehensive promotion initiative. This would clear the confusion in the minds of lay persons and lead to augmentation of the response. Fischer *et al*. have also emphasized the importance of coordination with other organ donor programmes.[13] It was observed that skin allograft donation by living donor was not promoted or recommended anywhere in the world except by some burn care professionals in our country. Allografts from live donor have no advantage over those obtained after death. The need for pre-harvest investigations, anaesthesia, surgery, pain, wound healing issues, scarring, hospitalization and limitation on the usable donor area does not justify the encouragement of this method of procuring skin allografts. It would be prudent for all of us to make concentrated co-operative efforts to promote skin donation from deceased donor.

Deep burn injuries preclude the ability of the skin to regenerate and heal. Paucity of autograft donor area or unsuitability of the wound for autografting may require temporary use of skin substitutes to promote wound healing, reduce pain and prevent infection or till re-harvesting of donor sites becomes possible. These substitutes include deceased donor skin allograft, xenograft, cultured epithelial cells and biosynthetic skin substitutes. The beneficial effects of skin allografts have been documented by many from Germany,[7] Holland,[8] U.K.,[14] U.S.A.[15] and Singapore.[16] Over the past 10 years, we have used skin allografts from 240 donors for 158 recipients, 7 with non-burn soft tissue loss and 151 with burn injury. Allografts were utilized for providing temporary long-term wound closure following primary burn wound excision in 48 (31.79%) patients and for infection control and wound bed preparation in 43 (28.48%) patients [Table 6]. Out of 23 patients with poor general condition, 21 were received in transfer from other healthcare facilities. It was observed that the skin allografts provided excellent pain relief and reduced the autograft requirement due to promotion of epithelialization, improved general condition and nutritional status significantly and achieved infection control and remarkable wound bed improvement with 100% autograft take in 40 patients (there were 3 deaths in this group). Eighty out of 84 patients with burn extent greater than 50% TBSA survived [Table 7]. This includes three patients from two mass casualty incidents with burn extent ranging from 67 to 85% TBSA. Total seven out of 151 patients expired. We are convinced that many more would have died if we did not have the facility for allograft skin banking.

It is amazing to note that even a small country like Finland has a multi-organ donation programme with facility for allograft skin banking.[17] However, despite a large number of burn patients with large extent of burn, ours was the only allograft skin bank striving to promote deceased donor skin donation in our country till mid 2008. Successful functioning of an allograft skin bank demands constant vigilance, quality control and a team of dedicated members. All involved need to understand and honour the ethical and moral significance of this donation after death that can give a gift of life to a burnt patient. All of us involved in burn care know that most of our patients are in prime of life and with healthy pre-morbid status. We all need to join hands to ensure that we save as many lives as possible and help their meaningful return to the family and society. It is not difficult to establish one skin bank in one city, preferably affiliated to a medical college burn centre. Our experience shows that this decreases the expenses on infrastructure and personnel very significantly. Presence of surgical trainees provides a pool of experts for allograft procurement. Bigger metropolises like Mumbai also need collection centres in different parts of the city to curtail the response time. The quality of allografts can be further improved with the use of electric dermatome. Like-minded, burn care professionals should come together to formulate guidelines and to provide assistance in establishment of new skin banks as well as quality control.

## CONCLUSION

The first deceased donor skin allograft bank in India was established on 24^th^ April 2000 at Lokmanya Tilak Municipal medical college and hospital, Sion, Mumbai. Till 31^st^ March 2010, it had received allograft skin donation from 262 donors. Allografts from 249 donors were used for 165 patients during this period. The encouraging response of the society to this concept of skin donation after death and the obvious benefit the patients received from skin allografts strongly suggests that we should establish more skin banks in the country to improve survival probability of patients with large burns.

Note: Skin bank manual is available on request at Department of Surgery, LTM Medical College and Hospital, Sion, Mumbai – 400 022.
